# Supplementary material for: Neuroprotection of NRF2 against Ferroptosis after Traumatic Brain Injury in Mice
Source: Antioxidants (Basel). 2023 Mar 16;12(3):731. doi: 10.3390/antiox12030731 (PMC10044792; doi:10.3390/antiox12030731)
Supplement: Supplementary file 1 [file antioxidants-12-00731-s001.zip › antioxidants-2234624-supplementary.pdf]

Supplementary materials

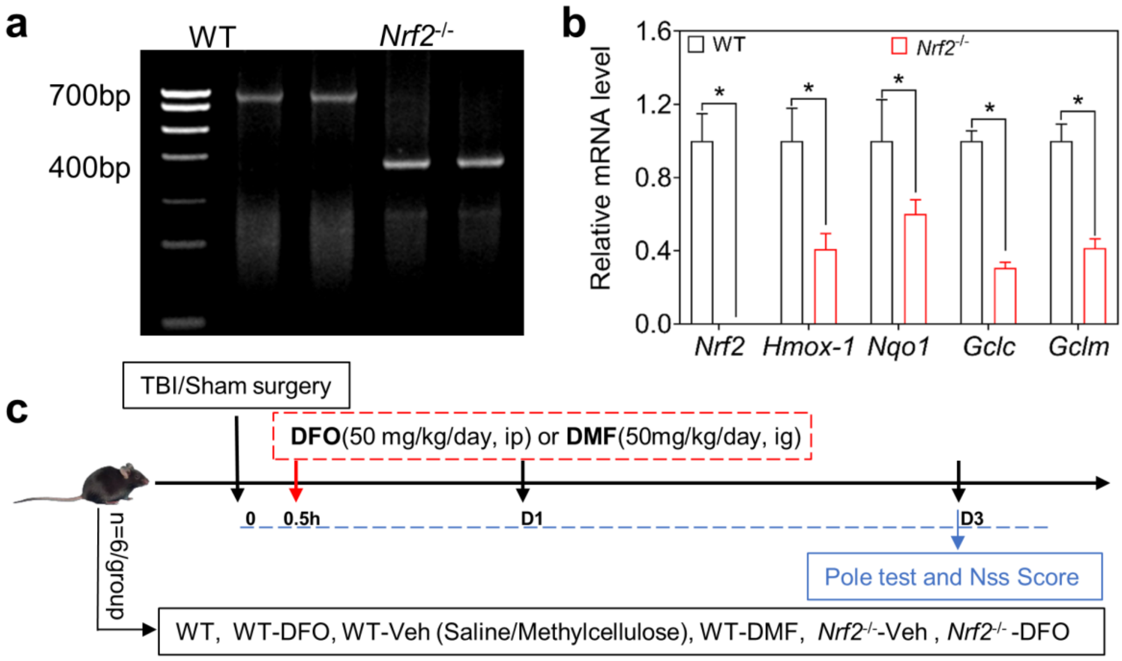

Supplementary Figure S1 Experimental protocol for traumatic brain injury (TBI) and genotype validation of *Nrf2*$^{-/-}$ mice

(a) Representative images of PCR for mouse genotyping. **(b)** mRNA levels of *Nrf2*, *Ho1*, *Nqo1*, *Gclc*, and *Gclm* mRNA, normalized to *β-Actin*, Data are expressed as mean ± SD, n = 3, * $p < 0.05$ vs WT, Student *t*-test for comparison of two groups. **(c)** The schematic flow diagram for grouping and treatment of mouse in TBI model, n = 6/subgroup.

WT, wild-type; SD, standard deviation

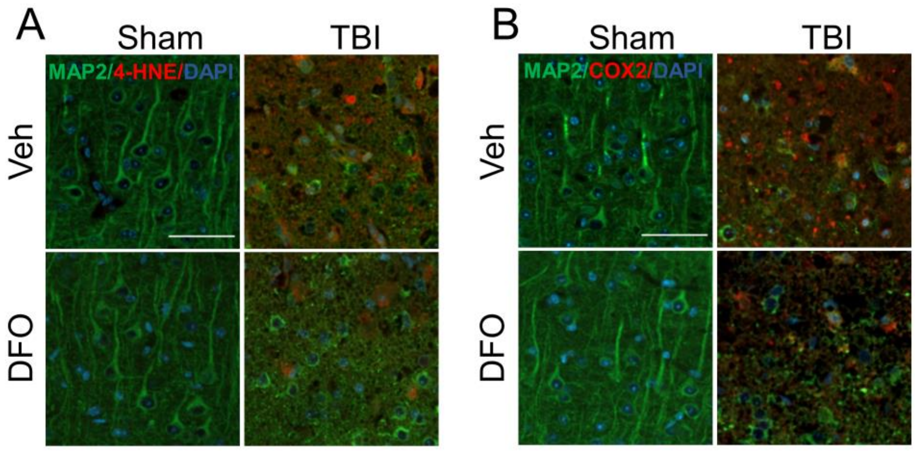

Supplementary Figure S2 Expression of 4-HNE and COX2 in neurons

(a-b) Representative double immunostaining images of MAP2 with 4-HNE **(a)**, COX2 **(b)** in injured cortex of WT mice with Veh or DFO treatment at 3dpi. Bar = 50 μm, n = 3.

Veh, Vehicle; DFO, deferoxamine; WT, wild-type; dpi, days post-injury

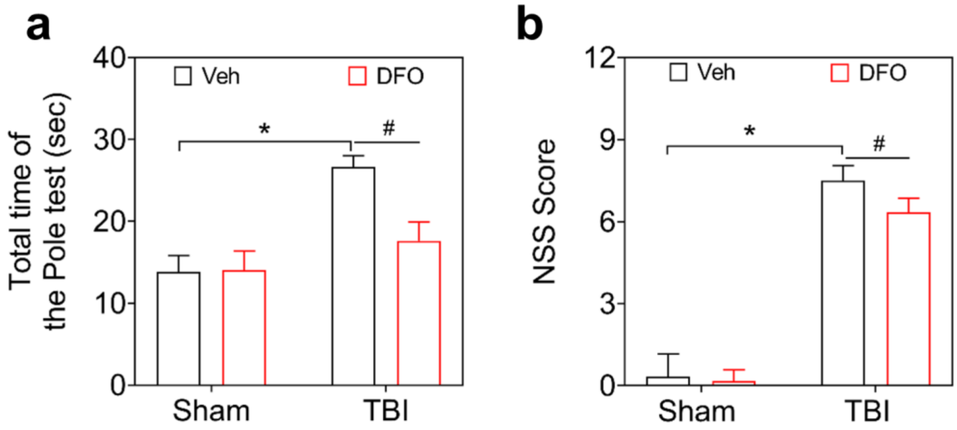

Supplementary Figure S3 DFO improves motor function and spatial memory in mice after TBI

(a) Time spent on the Pole Test at 3 dpi in WT mice. Data are expressed as mean ± SD, n = 6, * $p < 0.05$ vs Sham-Veh, # $p < 0.05$ vs TBI-Veh. **(b)**NSS of wild-type (WT) mice at 3 dpi. Data are expressed as mean ± SD, n = 6, * $p < 0.05$ vs Sham-Veh, # $p < 0.05$ vs TBI-Veh. Two-way analysis of variance followed by a Tukey post hoc multiple comparison test for comparisons more than two groups.

DFO, deferoxamine; TBI, traumatic brain injury; WT, wild-type; Veh, vehicle; dpi, days post-injury; NSS, neurological severity score; SD, standard deviation

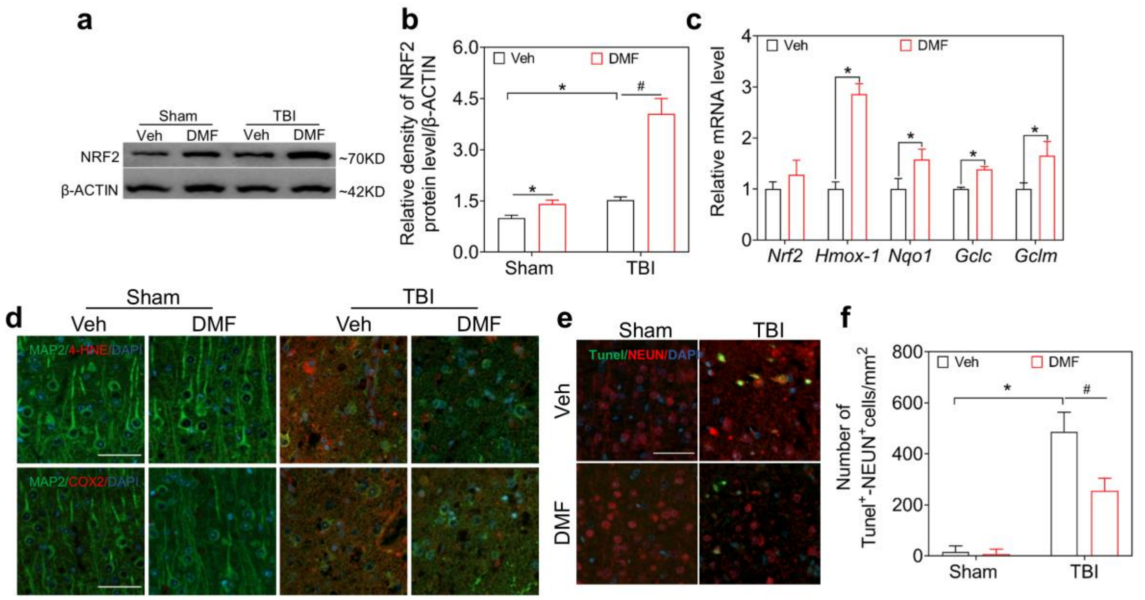

Supplementary Figure S4 DMF activates the expression of NRF2 protein, *Nrf2*-related downstream genes of in WT mice, and reduces 4-HNE, COX2, and TUNEL positive neurons

(a) Representative protein levels of NRF2 in the injured cortex of WT mice in the Veh or DMF-treated group at 3 dpi. **(b)** Relative intensity of NRF2 in injured cortex of WT mice in the Veh- or DMF-treated group at 3 dpi. n = 3. Data are expressed as mean ± SD, n=6, * p < 0.05 vs Sham-Veh, $^{\#}$ p < 0.05 vs TBI-Veh. **(c)** Expression of *Nrf2* and its downstream genes *Ho1, Nqo1, Gclc* and *Gclm* in injured cortex of the WT mice treated with Veh or DMF at 3 dpi, normalized to *β-Actin.* Data are expressed as mean ± SD, n = 6, * p < 0.05 vs Veh. **(d)** Co-staining of MAP2 with 4-HNE and COX2 in injured cortex of WT mice treated with Veh or DMF at 3 dpi. Bar = 50 μm. **(e)** Co-staining of TUNEL and NeuN in the injured cortex of WT mice treated with Veh or DMF at 3 dpi. Bar = 50 μm. **(f)** Diagram of TUNEL-NeuN-positive cells in injured cortex of WT mice treated with Veh or DMF at 3 dpi. Data are expressed as mean ± SD, n = 3, * p < 0.05 vs Sham-Veh, $^{\#}$ p < 0.05 vs TBI-Veh. Two-way analysis of variance, followed by a Tukey post-hoc multiple comparison test, was used for comparisons including more than two groups, and Student *t*-test for comparison of two group.

DMF, dimethyl fumarate; WT, wild-type; Veh, vehicle; SD, standard deviation; TBI, traumatic brain injury

Supplementary Figure S5 Original figures of Western Blot.

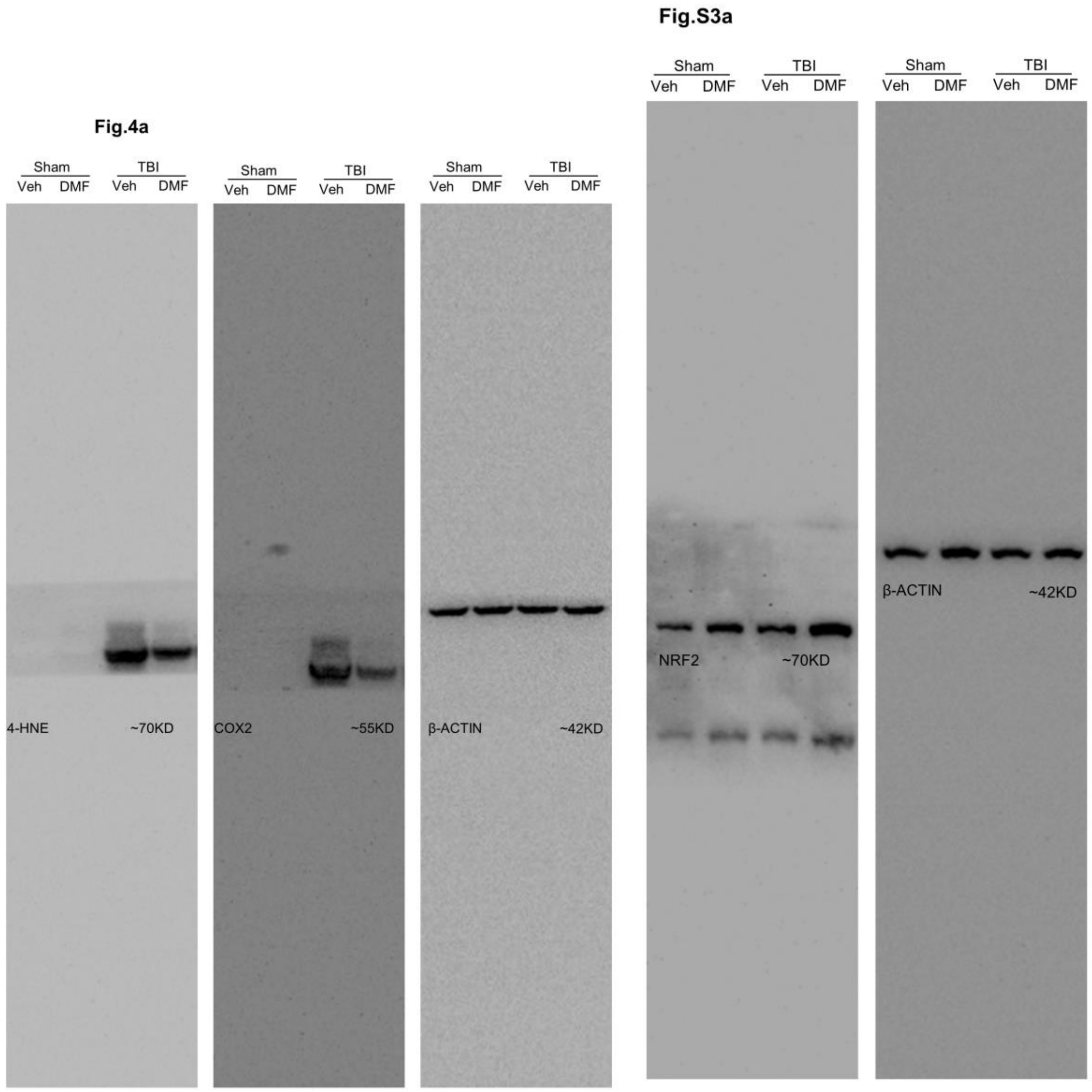

Fig.5a

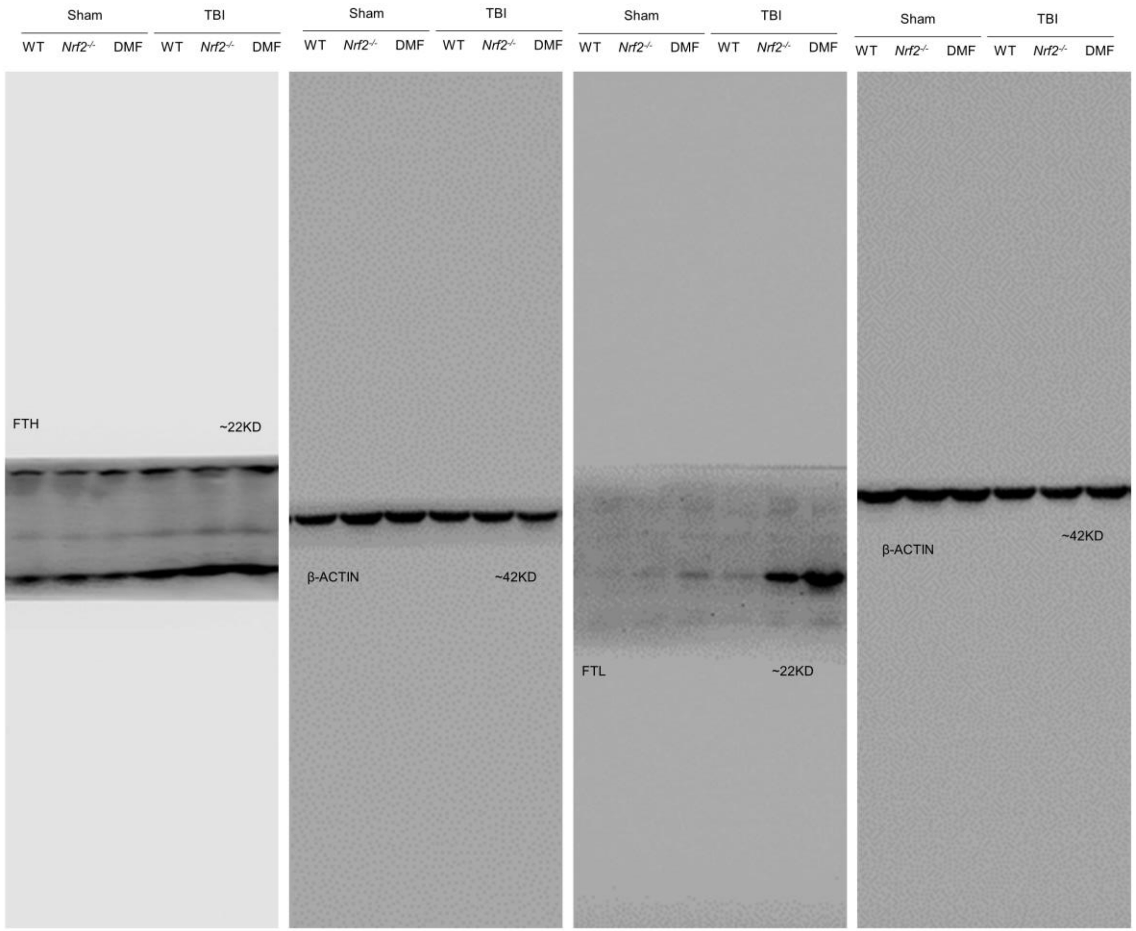
Sham
TBI
WT
Nrf2-/-
DMF
FTH
~22KD
β-ACTIN
~42KD
FTL
~22KD
β-ACTIN
~42KD

Fig.5a

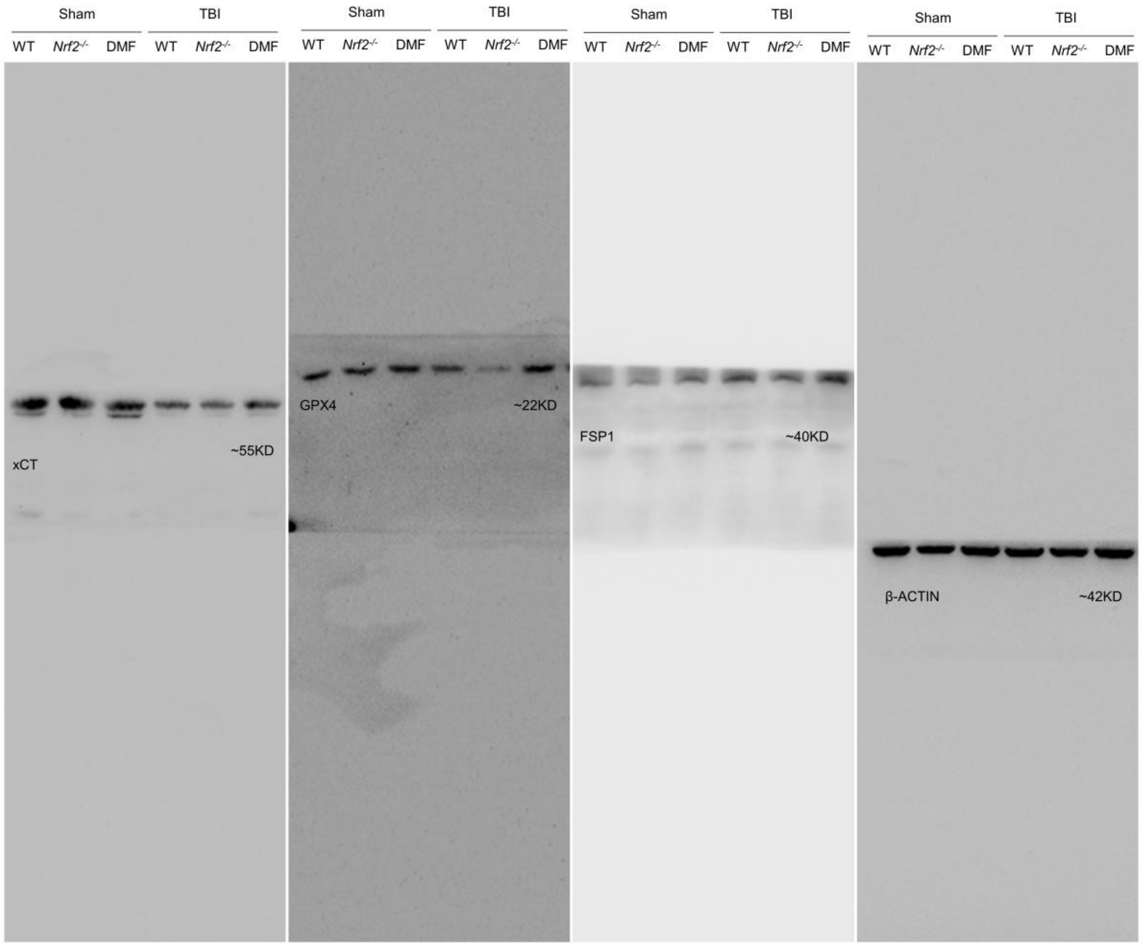
Sham
TBI
WT
Nrf2-/-
DMF
WT
Nrf2-/-
DMF
xCT
~55KD
GPX4
~22KD
FSP1
~40KD
β-ACTIN
~42KD

Supplementary Table S1 Antibodies used in this research

Antibody	Host	Distributor	Cat.NO	Dilution
**Primary antibody**				
MAP2	Rabbit	Proteintech	17490-1-AP	1:400 (IF)
FTH	Rabbit	Abcam	ab75973	1:1000 (WB)
FTL	Rabbit	Proteintech	10727-1-AP	1:1000 (WB)
GPX4	Rabbit	Proteintech	67763-1-Ig	1:1000 (WB)
FSP1	Rabbit	Proteintech	20886-1-AP	1:1000 (WB)
xCT	Rabbit	Abcam	ab175186	1:1000 (WB)
NRF2	Rabbit	Abcam	ab31163	1:1000 (WB)
NeuN	Mouse	Millipore	MAB377	1:400 (IF)
β-ACTIN	Mouse	Proteintech	23660-1-AP	1:1000 (WB)
COX2	Mouse	Proteintech	66351-1-Ig	1:400 (IF)<br>1:1000 (WB)
4-HNE	Mouse	R&D system	MAB3249	1:400 (IF)<br>1:1000 (WB)
**Secondary antibody**				
Donkey anti-Mouse IgG, Alexa Fluor 594	Donkey	Thermo Scientific	A-21203	1:400
Donkey anti-Rabbit IgG, Alexa Fluor 488	Donkey	Thermo Scientific	A-21206	1:400
Goat anti-Mouse IgG	Goat	ZSGB-BIO	ZB-2305	1:1000 (WB)
Goat anti-Rabbit IgG	Goat	ZSGB-BIO	ZB-2301	1:1000 (WB)

Supplementary Table S2 Primers sequences used in this research

Gene name	Primer sequence
Fth	Forward:5'- CTTTGCAACTTCGTCGTTCC -3'
	Reverse:5'- AGGTTGATCTGGCGGTTG -3'
Ftl	Forward:5'- CTCTGGGCGAGTATCTCTTTG -3'
	Reverse:5'- AGTGGCTTGAGAGGTTCATTC-3'
Ptgs2	Forward:5'- CTCACGAAGGAACTCAGCAC -3'
	Reverse:5'- GGATTGGAACAGCAAGGATTTG -3'
Acsf2	Forward:5'- AGATGAACATGAAGGAGCTGG -3
	Reverse:5'- ATAATTCTGCCCACACTACCAG -3
Fsp1	Forward:5'- GAGTACATCAAGGTGGAGACAG -3
	Reverse:5'- TCGTTCACTTTCAGAGCACC -3
β-Actin	Forward:5'- TCCTTCCTGGGCATGGAG -3
	Reverse:5'- AGGAGGAGCAATGATCTTGATCTT -3
Gclm	Forward:5'- AATCAGCCCCGATTTAGTCAG -3
	Reverse:5'- CGATCCTACAATGAACAGTTTTGC -3
Gclc	Forward:5'- ACCATCACTTCATTCCCCAG -3
	Reverse:5'- TTCTTGTTAGAGTACCGAAGCG -3
Nqo1	Forward:5'- TGAAGAAGAGAGGATGGGAGG -3
	Reverse:5'- GATGACTCGGAAGGATACTGAAAG -3
Ho1	Forward:5'- ACAGAGGAACACAAAGACCAG -3
	Reverse:5'- GTGTCTGGGATGAGCTAGTG -3
Nrf2	Forward:5'- TCCCATTTGTAGATGACCATGAG -3'
	Reverse:5'- CCATGTCCTGCTCTATGCTG -3'
